# Unique genes in plants: specificities and conserved features throughout evolution

**DOI:** 10.1186/1471-2148-8-280

**Published:** 2008-10-10

**Authors:** David Armisén, Alain Lecharny, Sébastien Aubourg

**Affiliations:** 1 Unité de Recherche en Génomique Végetale (URGV), UMR INRA 1165 – CNRS 8114 – Université d'Evry Val d'Essonne, 2 rue Gaston Crémieux, CP 5708, F-91057 Evry Cedex, France

## Abstract

**Background:**

Plant genomes contain a high proportion of duplicated genes as a result of numerous whole, segmental and local duplications. These duplications lead up to the formation of gene families, which are the usual material for many evolutionary studies. However, all characterized genomes include single-copy (unique) genes that have not received much attention. Unlike gene duplication, gene loss is not an unspecific mechanism but is rather influenced by a functional selection. In this context, we have established and used stringent criteria in order to identify suitable sets of unique genes present in plant proteomes. Comparisons of unique genes in the green phylum were used to characterize the gene and protein features exhibited by both conserved and species-specific unique genes.

**Results:**

We identified the unique genes within both *A. thaliana *and *O. sativa *genomes and classified them according to the number of homologs in the alternative species: none (U{1:0}), one (U{1:1}) or several (U{1:m}). Regardless of the species, all the genes in these groups present some conserved characteristics, such as small average protein size and abnormal intron number. In order to understand the origin and function of unique genes, we further characterized the U{1:1} gene pairs. The possible involvement of sequence convergence in the creation of U{1:1} pairs was discarded due to the frequent conservation of intron positions. Furthermore, an orthology relationship between the two members of each U{1:1} pair was strongly supported by a high conservation in the protein sizes and transcription levels. Within the promoter of the unique conserved genes, we found a number of TATA and TELO boxes that specifically differed from their mean number in the whole genome. Many unique genes have been conserved as unique through evolution from the green alga *Ostreococcus lucimarinus *to higher plants. Plant unique genes may also have homologs in bacteria and we showed a link between the targeting towards plastids of proteins encoded by plant nuclear unique genes and their homology with a bacterial protein.

**Conclusion:**

Many of the *A. thaliana *and *O. sativa *unique genes are conserved in plants for which the ancestor diverged at least 725 million years ago (MYA). Half of these genes are also present in other eukaryotic and/or prokaryotic species. Thus, our results indicate that (i) a strong negative selection pressure has conserved a number of genes as unique in genomes throughout evolution, (ii) most unique genes are subjected to a low divergence rate, (iii) they have some features observed in housekeeping genes but for most of them there is no functional annotation and (iv) they may have an ancient origin involving a possible gene transfer from ancestral chloroplasts or bacteria to the plant nucleus.

## Background

The role of gene duplications in evolution was suggested forty years ago (see the review by Taylor and Raes 2004 [1]). More recently, complete sequencing of several eukaryotic genomes showed the quantitative importance of duplicated genes [2,3]. In particular, plant genomes contain a high proportion of duplicated genes and, in several plant gene families, the number of paralogous genes is more than one hundred [4,5]. Frequent gene duplications [6], occasional segmental [7], chromosomal and genomic duplications [8-13] shaped present genomes. The underlying mechanisms indicate that the primary molecular events in gene duplication should affect most of the genes independently of their function. Nevertheless, all characterized genomes include single-copy (unique) genes, *i.e*. genes without apparent homolog in the same genome [14] and, for some of them, without any homolog, even in phylogenetically close relatives [15]. Indeed, evolution is not a one-direction process and a high proportion of duplicated genes are rapidly lost [6,16,17]. This definition of unique gene is fully independent of the gene function and is only based on the protein sequence uniqueness in the whole proteome of a considered species. For instance, in the framework of this definition, the bHLH transcription factors, whatever the different functions that might be assigned to each of them, are not considered as unique because they all share sequence similarity and, as such, are thought to have arisen from a common ancestor. In other words, in this paper we define as single-copy or unique gene, a gene coding for a protein without detectable sequence motif or global similarities with any protein in the same proteome.

Unlike gene duplication, gene loss is not an unspecific mechanism but it is instead influenced by functional selection [12,18]. Thus, duplicates that are maintained show a bias toward certain gene functional classes [19] or transcriptional level [6,20,21]. Unique genes may also be duplicates that diverged too much to be distinguished now [22]. With the recent availability of whole plant proteomes it is possible to consider further some questions about the generation and evolution of unique genes in plants. In many evolutionary studies, sound groups of duplicated genes are selected but the genes left apart by the process are far from being all unique genes. Indeed, the potential adaptive significance of duplicated genes and genomes has received great attention [23-25]. It is however more difficult to speculate on the meaning of species- or phylum-specific unique genes mainly because of a critical lack of functional annotation for most of them. Major differences in gene repertoire among species were attributed to proteins with obscure features that lack currently defined motifs or domains (POFs) and are often species- or phylum-specific [26]. The definition of POFs [27] relying only on the absence of characterized conserved sequence signatures is thus independent of the existence or absence of paralogs. POFs and unique genes are nevertheless overlapping populations of genes. Hypotheses on the possible origins of POFs include convergent evolution and rapid divergence [26]. The question of the origin of unique genes, either purifying selection against duplicates or rapid divergence, remains unsolved. In this study we first established and used stringent criteria in order to identify suitable sets of unique genes present in the extensively known proteomes of *Arabidopsis thaliana *(core eudicotyledons, *Brassicaceae*) and *Oryza sativa *(*Liliopsida*, *Poaceae*), two plants that diverged ~150 million years ago (MYA) [28,29]. Second, we used the intersection between the two sets of unique genes in order to characterize a set of genes conserved as unique in both *A. thaliana *and *O. sativa*, *i.e*. pan-orthologs as defined by Blair *et al*. [30]. Third, we searched for gene, promoter and protein features shared between all unique genes and/or within pairs of pan-orthologs. Fourth, using the pan-orthologs between *A. thaliana *and *O. sativa*, we searched for their conservation in a green unicellular alga and a moss, for which reasonably good proteomes are also available. Within the limits of the proteomes used, we show that several unique genes are species specific but that a significant number are conserved even outside of the green phylum. The clusters of homologous unique genes highly conserved throughout the green phylum globally present specific structural features that indicate a strong purifying selection supporting the orthology links between the conserved unique genes. These conserved unique genes would be important targets for functional studies since it is likely that they perform ancient but not described biological functions.

## Results and discussion

### How many unique genes in *Arabidopsis thaliana *and *Oryza sativa*?

**Figure 1:**
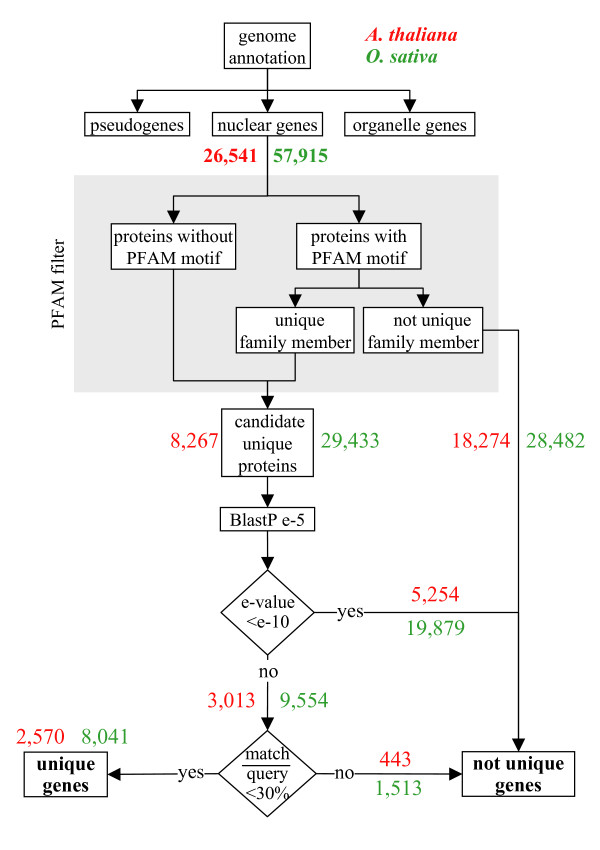
**Characterization of unique genes in *A. thaliana *and *O. sativa***. Schematic diagram describing the different filters applied to obtain the list of unique genes in each species. Only the proteins encoded by the nuclear genes were used. PFAM filter removed members of known families and BLASTp filters eliminated other genes with at least one homolog in the same genome. Results from *A. thaliana *genome are labelled in red while *O. sativa *results are in green.

With the scope to search for possible evidence of particular features of the unique proteins, our method should be stringent enough to deliver a minimum level of false positives. To achieve this objective we used a protocol that mixed detection of conserved motifs (through the PFAM library [31]), and local sequence alignments (BLASTp) taking into account the relative length of the conserved regions. *A. thaliana *and *O. sativa *were the first two plants with a whole genome sequenced and annotated [4,5]. The corresponding proteins have been used separately to run our protocol for each species (Figure 1). In a first stringent step, we removed 18,274 *A. thaliana *and 28,482 *O. sativa *proteins tagged with the same PFAM motifs. In a second step, remaining proteins were used as query sequence in a BLASTp search [32] against their corresponding proteome. Proteins that returned a hit with an e-value higher than e-10 were filtered on the basis of size ratio value of the best alignment between both proteins. This third step led us to consider as homologs, and thus not unique, proteins sharing low sequence similarities that are distributed on more than 30% of the full-length protein. Following this pipeline, we found 2,570 unique proteins in the proteome of *A. thaliana *and 8,041 unique proteins in *O. sativa*, which represent 9.7% and 13.9% of the whole proteome respectively.

Previous published estimations of the number of *A. thaliana *unique proteins gave different values ranging from 3,405 to 12,265 proteins [4,33-35] depending on the protocol used. The smaller value (3,405) comes from the PHYTOPROT project [34] and were obtained through extensive all-against-all sequence comparisons using the LASSAP software [36]. The list of unique genes delivered by PHYTOPROT was longer than the list provided by our method but 81% of the unique proteins were shared between both lists. The expertise of additional proteins identified in PHYTOPROT shows that they are members of a PFAM family and, therefore, excluded from our list.

### Unique proteins conserved and non-conserved between *Arabidopsis thaliana *and *Oryza sativa*

One protein unique in a given species may have either no, one or several homologs in other species. We named U{1:0} the unique proteins in one species with no homolog in the other one, U{1:1} the unique proteins with only one homolog and U{1:m} the unique proteins with more than one homolog. A 2-letter prefix was added to indicate the plant species when necessary, *i.e*. AtU{1:m} refers to *A. thaliana *unique genes with at least 2 homologs in the *O. sativa *genome. Both U{1:1} and U{1:m} are conserved single copy genes in the reference genome (thereafter called conserved single copy genes) and are respectively qualified as pan-orthologs and syn-orthologs according to Blair *et al*. [30].

**Figure 2:**
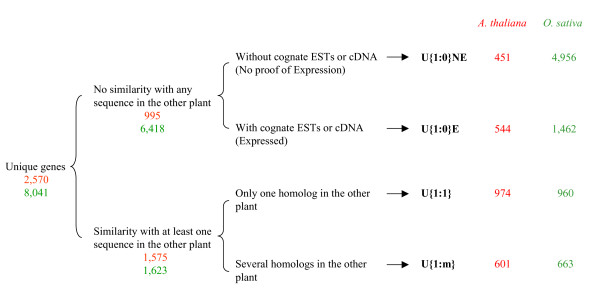
**Unique gene classification**. Based on BLASTp sequence comparison, *A. thaliana *and *O. sativa *unique genes were classified according to the number of homologs in the other species. We named U{1:0} the unique proteins in one species with no homolog in the other one, U{1:1} the unique proteins with only one homolog and U{1:m} the unique proteins with more than one homolog. First, a BLASTp between unique protein in each species and the whole proteome of the other species was used to define U{1:0}, U{1:1} and U{1:m} gene groups. Proofs of transcription (presence of cognate ESTs and/or cDNA) were used for further classification of U{1:0} genes in U{1:0}E (for Expressed) and U{1:0}NE (for No proof of Expression) genes. Red numbers are relative to *A. thaliana *while green ones are relative to *O. sativa*.

After sequence comparison based on BLASTp, 995 (3.7% of the whole *A. thaliana *proteome) and 6,418 (11.1% of the whole *O. sativa *proteome) unique genes were classified as AtU{1:0} and OsU{1:0} respectively (2). Sequence conservation between the *Liliopsida *and core eudicotyledon members of a pair of proteins is a strong support for the gene prediction of U{1:1} and U{1:m} genes. However, an over-prediction of U{1:0} genes remained possible. Thus, we searched for proofs of transcription for the genes coding for the U{1:0} proteins in both plants. We have found transcript sequences for 544 (out of 995) and 1,462 (out of 6,418) U{1:0} proteins from *A. thaliana *or *O. sativa *respectively. This class of proteins for which the corresponding gene structure was sustained by transcript sequences (ESTs and/or cDNA) was named U{1:0}E (for Expressed) genes. Similarly, the class of unique proteins without homologs in the other plant species and without cognate ESTs was named U{1:0}NE (for No proof of Expression) genes (Figure 2).

In *A. thaliana*, we further analysed possible over-prediction of 451 AtU{1:0}NE proteins searching for corresponding gene expression in CATMA [37] and Affymetrix [38,39] transcriptome resources. Statistical proof of expression was found for 311 additional AtU{1:0}NE genes. All together, these data indicated that most of the predicted AtU{1:0} coding genes were expressed and thus actual genes. It was more difficult to conclude on the accuracy of the number of unique genes for *O. sativa *since there remained a large number of OsU{1:0}NE genes (4,956) with not enough available transcriptome data.

Using the 2,570 *A. thaliana *unique proteins as query in a BLASTp against the 8,041 *O. sativa *unique proteins we found 974 pairs of AtU{1:1} proteins and 960 OsU{1:1} when doing the inverse search. Of these genes, 937 shared pairs remained as U{1:1}protein pairs after crossing both lists. A manual check of U{1:1} protein pairs present in only one list showed that differences were due to gene splitting/fusions that may come from either actual events or from gene prediction errors in one of the two genomes. These processes changed an actual U{1:1} relationship into an apparent U{1:m} relationship.

### Topological organization of unique genes

Both *A. thaliana *and *O. sativa *have large regions that are still recognizable as duplicated regions [4,40]. We analyzed AtU{1:0}, AtU{1:1} and AtU{1:m} gene distribution in *A. thaliana *non-duplicated regions, which contained 15.7% of the nuclear genome. No significant preferential occurrences of AtU{1:0}, AtU{1:1} and AtU{1:m} genes were observed inside the apparently non-duplicated regions, where we observed about 18% of them. Therefore, this result showed that most of the genes are unique not because they belong to a genomic region deleted after whole genome duplication, but because of the non-reciprocal local losses between two paralogous duplicated genomic regions.

We also analysed the distribution of each class of unique genes along *A. thaliana *and *O. sativa *chromosomes using a Chi-square test with a confidence level of 99.5% (critical values of 14.86 and 26.76 respectively). All gene classes were evenly distributed among the 5 chromosomes of *A. thaliana *with a Chi-square of 3.91 for U{1:0}, 3.95 for U{1:1} and 0.63 for U{1:m} genes. The *O. sativa *distribution was also even for U{1:0} and U{1:m}, chi-square of 23.63 and 25.64 respectively, but unequal (Chi-square of 65.10) on U{1:1} genes. Detailed analysis showed that in *O. sativa *genome there was a higher density of U{1:1} genes in chromosome 2 and 3 and a lower density in chromosome 11 and 12. This particular distribution is unexpected since chromosomes 11 and 12 are the only two rice chromosomes that do not show evidence for large regional duplications with any other rice chromosomes [40,41]. The recent duplication described between the first 3 Mb of the chromosomes 11 and 12 [13,41,42] only covers 11% of their size which is not sufficient to explain the low number of unique genes observed within each chromosome (60% of the expected number).

Thus, our results suggest that in *O. sativa*, as well as in *A. thaliana*, non-reciprocal losses between duplicated genomic regions are a frequent mechanism for generating and maintaining unique a set of genes.

### Unique gene and protein features

We compared the intron relative numbers, the presence of some TFBS and the protein lengths between random sets of nuclear genes and the 3 groups of unique genes, U{1:0}E, U{1:1} and U{1:m}. All the U{1:0}NE genes and the U{1:0}E genes not fully covered by cognate transcripts were not included in the study due to the uncertainty on their structural annotation (intron number and positions, CDS size). The GC content of all the groups was not significantly dissimilar to the 44.2% in *A. thaliana *and the 53.3% in *O. sativa*.

#### Intron number

This feature separates all the unique genes into two distinct groups. On one side, U{1:0} clustered intron poor genes that had 30% fewer introns than all nuclear genes. On the other side, U{1:m} and U{1:1} genes have a higher number of introns with a density of 1.35 and 1.57 introns per 100 amino acids as compared to 1.09 for all the nuclear genes in *A. thaliana *(Table 1). These differences are the same for rice unique genes. Our results are in agreement with the fact that, in general, evolutionarily conserved genes preferentially accumulate introns [43]. Nevertheless, there is no difference in the number of introns in the 5' and 3' UTRs between unique genes and the whole genome. These observations suggest that the pressure of selection that is at work to keep unique a set of orthologous genes in a genome has an effect down to the level of gene structures mainly in the ORFs. Indeed, functional reasons may be put forward since introns may play a functional role through alternative splicing, effects on gene expression [44,45] or by their involvement in protein transport [46].

**Table 1:** Features of unique genes and their promoter

	All other nuclear genes	U{1:0}E genes	U{1:1} genes	U{1:m} genes
***A. thaliana***				
Mean intron number	4.28	0.98	5.01	4.33
Mean protein size	392.88	133.53	318.05	318.75
Median protein size	352.00	107.00	262.00	249.50
Mean intron number/100 aa	1.09	0.73	1.57	1.35
TATA-box presence	18.8%	26.8%	10.3%	11.9%
TELO-box presence	10.9%	10.0%	15.2%	14.7%
SORLIP2-box presence	11.9%	14.1%	16.1%	15.2%
CAAT-box presence	26.2%	27.7%	34.9%	40.3%

***O. sativa***				
Mean intron number	3.85	0.85	4.89	4.10
Mean protein size	406.06	142.82	321.15	319.10
Median protein size	362.00	117.00	262.00	266.00
Mean intron number/100 aa	0.95	0.60	1.52	1.28
TATA-box presence	17.7%	16.9%	4.1%	7.0%
TELO-box presence	9.1%	6.4%	11.0%	12.9%
SORLIP2-box presence	38.1%	31.4%	41.2%	36.9%
CAAT-box presence	34.5%	33%	38.3%	31.2%

Only genes with CDS fully covered by transcripts (EST and/or cDNA) were used for the determination of intron numbers and protein sizes. TFBS in promoter regions were searched for only in promoters of genes with a UTR longer than 50 nucleotides as shown by at least one cognate transcript. The complete nuclear gene set minus the 3 classes of unique genes defines the "All other nuclear genes" class.

#### Transcription factor binding sites (TFBS) in promoter sequences

In the whole genome of *A. thaliana *and *O. sativa *we found respectively 20% and 16% of genes with a TATA-box in their promoters. Comparisons with the frequency of these two well characterized TFBS present in promoters of unique genes split them in two groups: the U{1:0} class on one side and the U{1:m} and U{1:1} classes on the other side. On one hand, the promoters of Arabidopsis U{1:m} and U{1:1} genes contains the same relative number of TATA-box (Chi-squared test, P-value = 0.40) and they have a significantly lower frequency of TATA-box (Chi-squared test, P-value = 2.3e-14) than the other nuclear genes (Table 1). On the other hand, TELO-box presence was significantly higher in AtU{1:m} and AtU{1:1}genes than in the other nuclear genes (Chi-squared test, P-value = 0.0057). The same differences are observed in unique *O. sativa *genes (Table 1). The two other TFBS analysed, SORLIP2 [47,48] and CAAT [49] boxes, present slight variations in each class when compared with whole genome distribution, but these variations were not consistent in both species (Table 1). The different frequencies of TATA and TELO boxes observed in the promoter sequences of unique genes cluster them as the intron density criteria: the class U{1:0} on one side and the two classes U{1:1} and U{1:m} on the other side. This particular clustering conserved in both *A. thaliana *and *O. sativa *is discussed below.

#### Protein length

**Figure 3:**
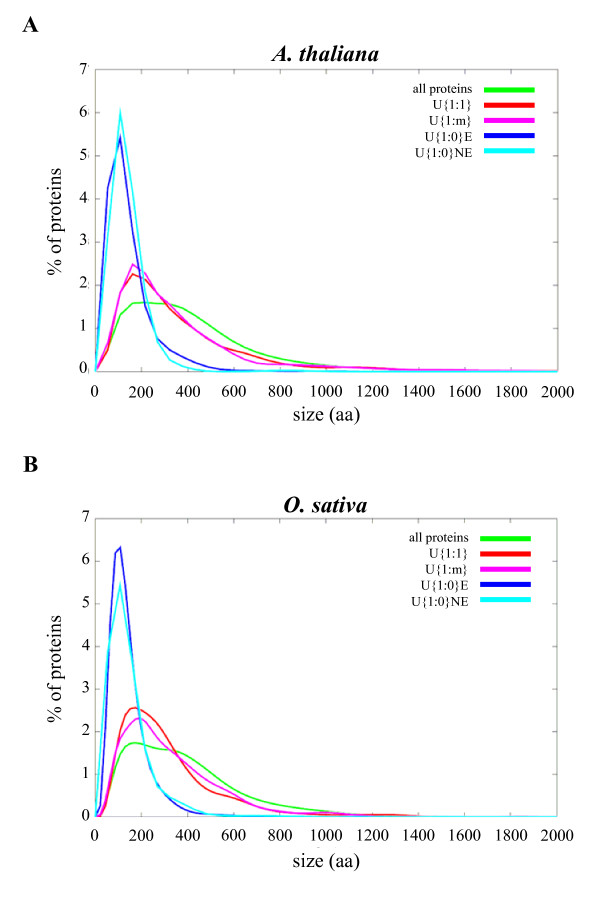
**Size distributions of proteins encoded by unique genes**. The size distributions of different groups of proteins encoded by unique genes are compared in *A. thaliana *(A) and *O. sativa *(B). The reference 'all proteins' corresponds to every proteins encoded by the nuclear genes.

We compared the size distribution of each group of unique proteins in the two species (Figure 3). On average, unique genes coded for shorter proteins than the whole genome. This is particularly evident for U{1:0} genes in both *A. thaliana *and *O. sativa *showing a mean length and a size distribution of the proteins smaller (Wilcoxon test, P-values < 2.2e-16) than in the other classes of unique genes (Figure 3). Indeed, the median size of the not unique *A. thaliana *proteins is 352 aa while the median value for the U{1:0}E proteins is only 107 aa, *i.e*. about 70% smaller (Table 1). While the displacement of the size distribution of unique genes towards the small values was shown in both plant genomes studied, it was less important in U{1:1} and U{1:m} proteins but still significant (Wilcoxon test, P-values < 1e-14). The size distribution of these two groups of conserved single copy genes had a maximum around 150 aa and is localized between the size distribution of U{1:0} proteins and the size distribution of the whole proteome (Figure 3). We may expect that the number of conserved single copy genes will increase in the near future since more genes coding for short polypeptides will be added to genome annotations. Indeed, the *ab initio *prediction of short ORFs is difficult [50-52] and recent results in *A. thaliana *show that a part of the drop in the size distribution of annotated gene products below 100 amino acids [53] may be due to the rejection by the annotation processes of several small ORFs that turned out to be transcribed and/or under purifying selection [54-56]. Similar situations have been reported in mouse, yeast and drosophila where experimental supports and comparative genomics indicate that many short ORFs code for functional elements involved in important biological processes such as cell signalling [57-59].

In summary, in the *A. thaliana *genome, there are 2,570 unique genes and 995 do not have a homolog in *O. sativa*. Conserved single copy genes are both the 974 *A. thaliana *genes that have only one ortholog and the 601 genes that have more than one homolog in *O. sativa*. In *O. sativa *genome, 8,041 genes are unique and 6,418 do not have a homolog in *A. thaliana*. Furthermore, 960 conserved unique genes have only one ortholog while 663 have more than one ortholog. Even if we might suspect some over-prediction of unique *O. sativa *genes, our results about the common features shared by unique genes are highly similar in both *A. thaliana *and *O. sativa*. First, conserved single copy genes (U{1:1} and U{1:m} classes) have relatively more introns than in the whole genome and their promoter is characterized by a lower presence of TATA-box and a higher presence of TELO-box than in the nuclear genes. Second, unique genes code for shorter proteins than the whole genome and the difference is the highest for unconserved proteins.

### Functional features of U{1:0} genes

We recovered the annotated gene functions available for the 544 AtU{1:0}E. Despite the fact that we used "annotation" in the largest acceptation of the word, only 105 of them have a predicted function (Table 2), *i.e*. 2 to 3 times less than expected from the whole genome [60]. In the 105 annotated AtU{1:0}E genes we observed 15 genes coding for recognized peptide phytohormones [61] including CLAVATA3 and 5 CLAVATA3 related peptides, POLARIS, 3 PROPEP, RALF and N Hydroxyprolin-rich glycoprotein coding genes. The small peptide phytohormones are involved in signalling roles in defence or non-defence functions [61] Most of the peptide phytohormones are proteolytic products of larger propeptides encoded by different genes. Some peptide phytohormones may be clustered based on short motif conservation such as CLAVATA3 group which is characterised by only 12 residues while the remaining parts of the propeptides are highly divergent. When we searched for peptide phytohormones in AtU{1:1} genes, we did not find any even though there were almost 6 times more genes with predicted functions compared to AtU{1:0}E genes. Another specific feature of the AtU{1:0}E group is to exhibit a relatively high percentage of genes coding for proteins targeted at the endoplasmatic reticulum (Table 2) as pro-peptides coding for secreted peptide phytohormones [61]. This observation suggests that the AtU{1:0}E group might contain many other not yet characterized genes coding for pro-peptides phytohormones and that might be involved in unknown signalling processes. For instance in the AtU{1:0}E group, we found 13 genes coding for proline or glycine rich-proteins that were mainly predicted to be targeted at the endoplasmic reticulum (Table 2). Additionally, genes encoding for secreted peptides have been reported as having a low intron density [53] as we observed for the U{1:0} group of genes.

**Table 2:** AtU{1:0}E and AtU{1:1} function comparison

			With predicted function		Peptide phytohormones		Pro- or Gly-rich proteins	
			
	Nb	ER (%)	Nb	ER (%)	Nb	ER (%)	Nb	ER (%)
AtU{1:0}E	544	19.7%	105	27.6%	15	46.7%	13	61.5%
AtU{1:1}	937	6.8%	610	6.0%	0	-	4	0.0%

Distribution of AtU{1:0}E and AtU{1:1} genes according their functional annotation and the presence of predicted targeting peptide to the endoplasmatic reticulum (ER).

### Structural and functional features conserved in At and OsU{1:1} gene pairs

The 937 pairs of U{1:1}genes between *A. thaliana *and *O. sativa *were established on local sequence comparisons (reciprocal best hit or RBH) of U{1:1} gene lists with criteria generally accepted to define an orthology relationship [62]. Nevertheless, to support more strongly the orthology and the functional relationships, we looked for some structural features shared by the two members of U{1:1} pairs (see Additional file 1).

#### Protein length

**Figure 4:**
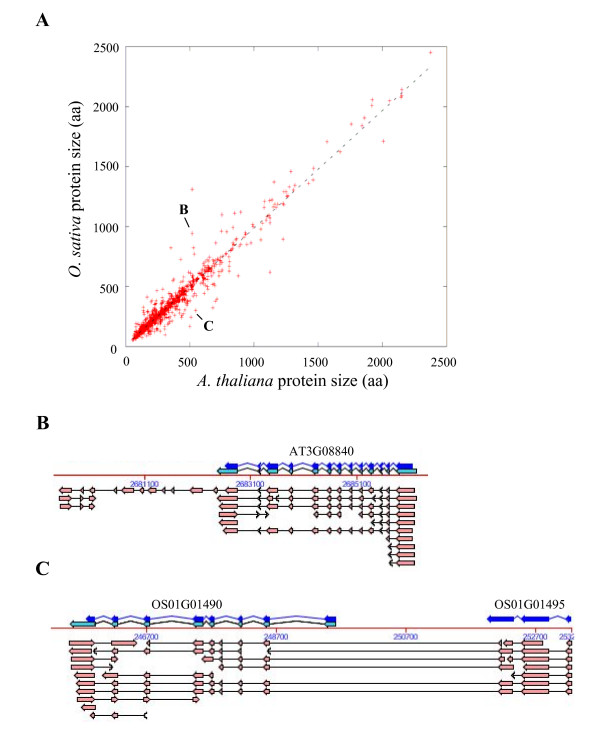
**Comparison of protein lengths in U{1:1} pairs**. Each point represents protein lengths (in aa) of one U{1:1} pair of proteins (A). The linear correlation between U{1:1} protein sizes is represented by a dotted line (r^2 ^= 0.94). Hand-checking of the largest differences showed that they are mainly due to erroneous predicted gene models with either an artificial exon gain/loss as in AT3G08840 (B) or a splitting/fusion process as in OS01G01490-OS01G01495 (C). Arrows and lines represent exons and introns while dark blue, light blue and pink colours represent predicted CDS, predicted mRNA and cognate transcripts (ESTs/cDNA), respectively. (B) and (C) are snapshots from FLAGdb^++ ^[90].

Protein lengths of the two members of a U{1:1} pair were highly correlated (Figure 4A) and the slope of the correlation was close to one. Indeed, 456 (49%) out of the 937 pairs had proteins with length differing by less than 5% of the total length. This high conservation in protein length between the proteins of a U{1:1} pair was also illustrated by the fact that in 526 pairs (56%) the difference between the two proteins was less than 20 amino acids. Nevertheless, a small number of U{1:1} pairs were more divergent with, for instance, 77 pairs (8%) showing differences in the protein lengths equal to or higher than 30%. We examined the 24 pairs exhibiting a length difference higher than 200 amino acids, and in 16 cases, the difference could be explained by errors in the predicted gene model of one of the two genes. In 4 out of 16 pairs we found an artifactual fusion or splitting of neighbour genes (Figure 4B) and in 12 out of 16 pairs the difference was due to an erroneous gain or loss of exons (Figure 4C) in one of the two species.

#### Intron position

The conceptual position of introns has been searched in the global alignment of each pair of protein sequences. Nearly 45% of U{1:1} pairs had conserved number and positions of introns, while the mean value for random pairs of conserved unique genes was 0.2% (Table 3). Less stringently, 71% of the U{1:1} pairs exhibited at least one intron at a conserved position as compared to 10.6% in the random pairs. Overall, the high intron conservation is strong evidence for orthology between members of a U{1:1} gene pair, discarding any mechanism of convergence between their sequences. Comparison of gene structures in the U{1:1} pairs also highlights the fact that, since the speciation, the numbers of intron gains or losses are nearly equivalent in the two species. Indeed, the ratio between the number of not conserved introns (in terms of position) in *A. thaliana *and the number of not conserved introns in *O. sativa *is 1.03 (Table 3). Comparative studies on *A. thaliana *and *O. sativa *genes showed three different evolutionary trends based on the orthology relationships. First, recent duplicated genes are submitted to high loss and gain of introns [63], second, two orthologous genes tend to keep the same gene structure and only a relatively small number of species-specific introns are observed [64] and, third, slowly evolving conserved genes are also subject to an elevated rate of intron gain but tend to conserve their introns [43]. As a consequence, there is a negative correlation between density of introns and sequence evolution rate of genes [43]. The density and the high conservation of intron positions in conserved unique genes, U{1:1}, suggests that these genes are orthologous and slowly evolving genes.

**Table 3:** Conservation of intron positions in U{1:1} gene pairs

	U{1:1} ortholog pairs	U{1:1} random pairs	Nuclear gene random pairs
Pairs with all conserved intron positions	44.9%	0.2%	0.1%
Pairs with no conserved intron position	44.4%	79.6%	58.9%
Pairs without any intron	3.7%	1.0%	5.1%
Pairs where only one gene has intron(s)	7.0%	19.2%	36%
Pairs with at least one conserved intron position	71%	10.6%	6.1%

Conserved intron number/total intron number in *A. thaliana*	60.5%	2.6%	1.7%
Conserved intron number/total intron number in *O. sativa*	59.6%	2.6%	1.9%
Number of not conserved introns in *A. thaliana*/not conserved introns in *O. sativa*	1.03	1.02	1.12

Intron position conservation was tested between 486 U{1:1} gene pairs (pairs in which both genes are supported by full-length transcript), and on random samples of 486 shuffled gene pairs from both species extracted fifty times from U{1:1} genes and from all nuclear genes. Intron position was based on the corresponding protein sequence alignments (ClustalW).

#### Transcription

**Figure 5:**
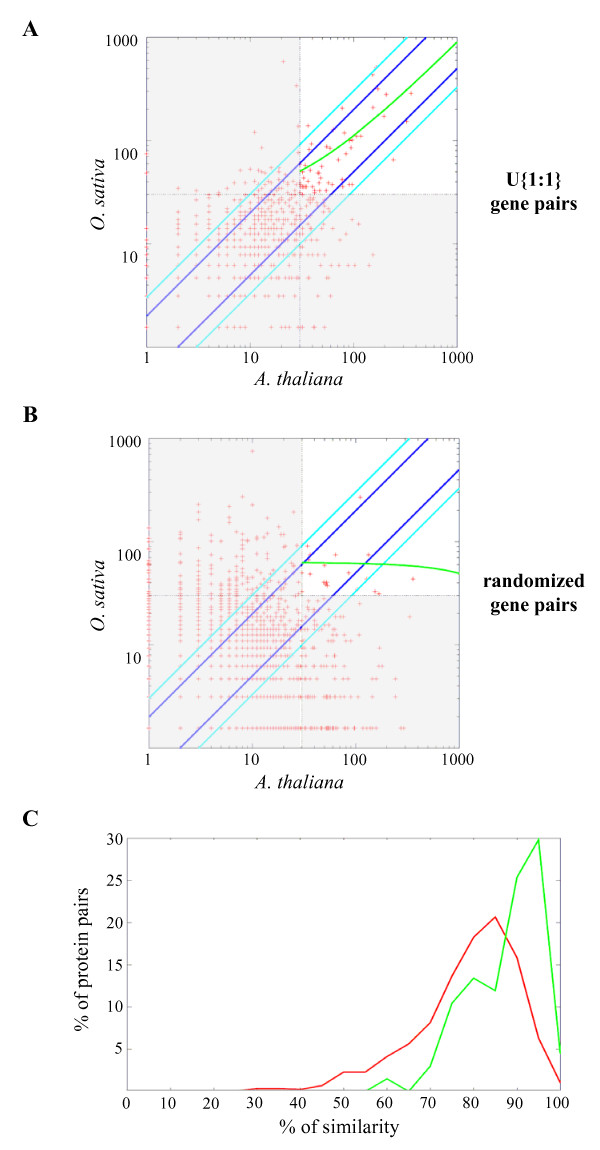
**Expression levels correlated between genes of U{1:1} pairs**. Expression level correlation based on the number of transcripts (ESTs/cDNA) associated to U{1:1} gene pairs (A) and randomized nuclear gene pairs (B). Values were first normalized to take into account the size of the transcript resources in each species, the number of genes with a transcript and the total number of genes on each species, and then transformed by base 10 logarithm. We used only the gene pairs with a size difference between proteins equal to or smaller than 20 aa (526 U{1:1} and 8,390 randomized pairs). The green line represents the linear correlation for pairs of genes with at least 30 cognate transcripts (white area). U{1:1} genes pairs: r^2 ^= 0.51 and Kendall's test P-value = 1e-6; Random pairs sample: r^2 ^= 0.03 and Kendall's test P-value = 0.26. Diagonal lines delimit an expression similarity of 33% (light blue) and 50% (dark blue). (C) Percentage of similarity was recovered from ClustalW alignments of U{1:1} protein pairs encoded by highly (green, more than 30 cognate transcripts) and lowly (red, less than 30 cognate transcripts) transcribed genes.

The methods available to compare the expression of orthologous genes from different species are limited. Since *A. thaliana *and *O. sativa *benefit from large collections of EST and cDNA sequences, we used the number of available cognate transcripts of each member of a U{1:1} pair to estimate and compare their expression levels. In order to avoid sampling bias, we focused our comparison on genes with at least 30 cognate transcripts. Retrieved information showed genes with at least 30 cognate transcripts are in similar proportion in the population of U{1:1} genes as in the whole genome whatever the considered species: 14.6% and 17.3% respectively for *A. thaliana *and 7.2% and 10.1% respectively for *O. sativa*. A correlation (Kendall's test, P-value = 1e-6) between the normalized numbers of transcripts in *A. thaliana *and *O. sativa *could be observed for U{1:1} pairs (Figure 5A). We compared this result with the correlation obtained with a random set of gene pairs having a maximum size difference of 20 amino acids to reflect U{1:1} size proximity. The random set contains ten times more gene pairs to compensate for the fact that associating not orthologous genes increases the chance of having at least one gene in the pair with less than 30 ESTs/cDNA. No correlation between the numbers of ESTs within the random set (Kendall's test, P-value = 0.26) was found (Figure 5B). Gene expression and evolutionary rate have been shown to be correlated in the genomes of different species [21,65,66] including plants [67]. Our results showed that this correlation held true for the limited set of conserved unique genes in *A. thaliana *and *O. sativa*. Indeed, similarities inside U{1:1} protein pairs coming from highly transcribed genes, i.e. with at least 30 cognate transcripts, were higher than similarities in the lowly transcribed U{1:1} pairs (Figure 5C). Therefore, the features expected for genes responsible for the same biological function, *i.e*. conservation both in sequence and in level of transcription as well as the positive correlation between them, are strongly observed between genes in U{1:1} pairs indicating their pan-orthology.

#### TFBS conservation

In the previous section, we showed that conserved unique genes have less frequently a TATA-box and more frequently a TELO-box in their promoters than the other genes. Nevertheless, the general over-representation of one TFBS in the unique gene promoter set does not mean that TFBS are conserved in the two promoters of pan-orthologs. Therefore, we searched for the number of simultaneous TATA-box or TELO-box presence on both promoters of each U{1:1} gene pair. Surprisingly, the percentage of pan-orthologs that presented a TATA-box motif within both promoters was only 0.8% and is not significantly different (Chi-squared test, P-value = 0.13) than the expected value, *i.e*. the value observed in promoters of randomly selected pairs of genes (0.4%). In contrary, the simultaneous presence of a TELO-box motif within both promoters of a U{1:1} pair was significantly higher (Chi-squared test, P-value = 5.22e-5) than found in random pairs (3.8% compared to 1.6%). In order to complete the promoter comparison between *A. thaliana *and *O. sativa *pan-orthologs, we used the CONREAL [68] and CREDO [69] packages to find any other conserved motifs, *i.e*. known or not known putative TFBS. This phylogenetic footprinting approach did not highlight a promoter sequence conservation different than that detected in random pairs of promoters. Additionally, the global analysis of all pan-ortholog promoter pairs with Motif sampler [70] failed to discover over-represented motifs excepted the previously identified TELO-box. Thus, contrary to our observation of conserved features in the CDS, we found almost no trace of sequence conservation within the promoters of U{1:1}gene pairs even if our dataset of pan-orthologs might be regarded as the best situation to see common regulatory sequences in *A. thaliana *and *O. sativa *promoters. Nevertheless, promoter pairs of pan-orthologs might share conserved TFBS (not over-represented in the unique gene population) which we cannot distinguish from background noise through the comparison of two sequences.

In summary, conserved genes maintained unique in both *A. thaliana *and *O. sativa *have (i) clearly a common origin as indicated by the conservation of the intron positions and the conservation in their product lengths, (ii) no apparent conservation between their promoters which contrasts with (iii) a conservation in their relative transcription level. Nevertheless, the number of ESTs that may be associated to a gene is a general indication of the level of transcription but it is a mixed measurement that is dependent on both high expression in specific situations and expression in a large range of conditions. Transcriptome data from DNA chips inform better on the breadth of expression. Analyses of large transcriptome data collections have shown that *A. thaliana *genes responding to many stimuli are frequently characterized by the presence of a TATA-box, shorter CDS and fewer introns [71,72]. Conversely, *A. thaliana *genes controlled by TELO-box have a narrow stimuli response and tend to be larger and have more introns [71]. In this context, the conserved single copy genes, which rarely contain a TATA-box and are relatively short genes containing more introns, might constitute a group of genes quite apart in the whole genome.

### Are unique *A. thaliana *and *O. sativa *genes conserved as unique in other plants?

**Figure 6:**
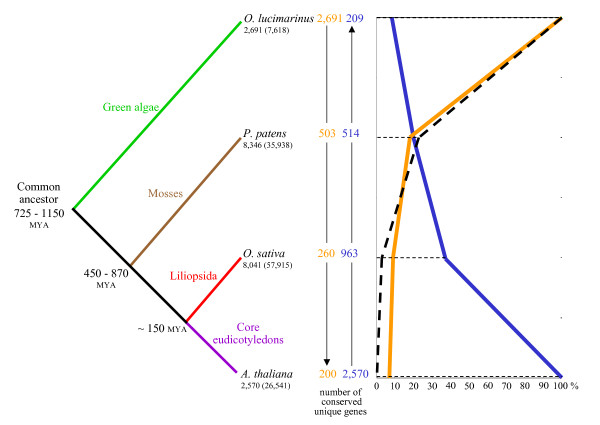
**Unique gene conservation in the plant kingdom**. Study of unique gene conservation through evolution of *Arabidopsis thaliana *(Brassicales), *Oryza sativa *(Poales), *Physcomitrella patens *(Funariaceae) and *Ostreococcus lucimarinus *(Prasinophyceae). Unique genes of each species were characterized (number below species name, total nuclear genes between brackets) and orthology relationships between couples of species were established using the previously described protocol. Phylogenetic conservation of unique genes was analysed from *O. lucimarinus *(orange line) and *A. thaliana *(blue line) discarding not conserved unique genes on each node (evolution distance showed in millions of years [28,29,74,78,79]. Remaining genes in each case were compared to eliminate inconsistencies and obtain a final list of 192 unique genes conserved as unique in the four species: U{1:1:1:1} genes. These 192 conserved unique genes are far more than the 8.38 U{1:1:1:1} genes expected by random conservation (black dashed line).

We extended our study to other genomes for which our knowledge was not as complete as for the *A. thaliana *and *O. sativa *ones but, nevertheless, with a relatively complete proteome available. Thus, we systematically searched, with our approach, for unique proteins in the available proteomes of *Ostreococcus lucimarinus *and *Physcomitrella patens *[73,74]. The nearly complete proteomes of *Populus trichocarpa *[75] and *Vitis vinifera *[76] were not used in our phylogenetic analysis in order no to distort our results by an overrepresentation of the core eudicotyledon branch. Two by two comparisons of the unique proteins from the 4 studied species showed that the number of U{1:1} pairs decreased with the evolutionary distance separating the plants. However, the numbers of the observed U{1:1} pairs were always significantly above the number expected by chance (Figure 6). There are about the same number of U{1:1} pairs, ranging from 477 to 503, between *O. lucimarinus *proteome and any one of the 3 other proteomes whatever their total number of proteins (ranging from 26,541 to 57,915). This result suggests that any of the multicellular plant genomes conserved about 500 of the unique 2,691 genes present in the unicellular *O. lucimarinus *genome. Globally, 53% of the 2,691 *O. lucimarinus *unique genes are also present in all or all but one species and 18% of the *O. lucimarinus *unique genes are also present in *P. patens *but not in the core eudicotyledon and *Liliopsida *plants used in the comparison. Similar results are obtained for *A. thaliana *unique genes with 48% of the unique genes present in all or all but one species, and 22% of *A. thaliana *unique genes only found in *O. sativa*.

The phylogenetic studies of unique gene conservation from *O. lucimarinus *to *A. thaliana *provided a final list of 192 unique genes, the intersection between the two lists (200 and 209) provided by comparisons going in the two opposite directions (Figure 6). We named as U{1:1:1:1} these genes conserved as unique in the 4 studied species. The 192 U{1:1:1:1} genes constitutes a particular subset (genes maintained as single copy in every studied species) of the 4,177 *A. thaliana *core genes defined as conserved in all plants by Vandepoele and Van de Peer [77]. The expected number of U{1:1:1:1}genes, if we assumed a random conservation between *O. lucimarinus *and *A. thaliana*, was only 8.38 genes (Methods section). The 192 genes present in all the studied species came from a common ancestor about 725–1,150 MYA [78,79] and have been conserved as unique in all the species despite numerous local and segmental duplications expected to have occurred during this long period of time [76]. In comparison, Zimmer *et al*. [78] have defined 26 pan-ortholog clusters but they have also considered *Cyanidioschyzon merolae *and *Pinus taeda *data and allowed for the exception that a single species might contain paralogs.

Structural features of U{1:1:1:1} genes showed a mean protein length and exon number similar to features in U{1:1} genes as well as the same tendency towards a low TATA-box and a high TELO-box presence in promoters. These characteristics suggest that unique genes underwent the same kind of selection pressure from the common ancestor to the present organisms. An estimation of this pressure was obtained by calculating the synonymous and non-synonymous substitution rates (dN and dS) with Nei-Gojobori's method [80] included in the Codeml program from the PAML package [81]. Each gene within a cluster of U{1:1:1:1} genes was paired and compared to every other gene included in the cluster (Table 4). Additionally, the dN/dS rate was computed for U{1:1} gene pairs. Results showed a high selective pressure against non-synonymous substitutions with a median dN/dS ratio of 0.32 for the 937 U{1:1} genes and from 0.25 to 0.41 for unique genes conserved among the three land plants and with a maximum median of 0.79 for pairs including *O. lucimarinus *(Table 4). In comparison, we observed that the median dN/dS ratio calculated from 7,551 alignments of putative *A. thaliana *– *O. sativa *orthologous proteins (RBH, Methods section) is 0.33. One dN/dS ratio of 1 is usually considered as the limit between a negative or a purifying selection (a drift being equal to 1 and a positive selection being higher than 1) [82,83]. Thus, our results show purifying selection pressure onto conserved unique genes in plants and strongly suggest that most of these genes are actual functional pan-orthologs.

**Table 4:** dN/dS rates in plant conserved unique genes

	*A. thaliana*	*O. sativa*	*P. patens*
*O. sativa*	0.25		
*P. patens*	0.41	0.33	
*O. lucimarinus*	0.79	0.73	0.72

Medians of synonymous and non-synonymous substitution rates (dN/dS) among all pairs in U{1:1:1:1} genes were calculated with Nei-Gojobori's method after ClustalW alignments.

### Phylogenetic conservation of unique genes and functional implications

The existence of homologs to U{1:1:1:1} genes in other species was searched by BLASTp against the Uniprot database in order to define the range of conservation in other branches of the tree of life. Our results show that 26% of U{1:1:1:1} genes were specific to plants, 13% were conserved in plants and bacteria, 43% could be found in both plants and metazoa, and 18% were conserved in all plants, bacteria and metazoa phyla. This phylogenetic profile shows that 74% of U{1:1:1:1} genes were highly conserved not only in plants but also in other life phyla. This situation implies an ancient origin of these genes and increases the probability for a critical function promoting their conservation. However, no evidence of shared or similar functions can be found in the fraction of U{1:1:1:1} proteins for which functional information has been inferred from sequence homologies. The fraction of unique conserved genes with a functional annotation, *i.e*. 60%, is the same as in all *A. thaliana *nuclear genes [60]. In order to get information about function and origin of unique plant genes, we explored the predicted subcellular localization of the proteins according to their phylogenetic profile (Table 5). This work was based on the analysis of the 937 U{1:1} proteins since the 192 U{1:1:1:1} proteins constitute too small a set to obtain statistically robust results. Compared to 20,000 random *A. thaliana *nuclear genes, the unique plant genes having homolog(s) only in bacteria frequently encode plastidial proteins since 49.1% of them have a predicted targeting peptide specific to chloroplasts (Table 5). We observed the same tendency within the 192 U{1:1:1:1} proteins. This significant bias (Chi-squared test, P-value = 1e-5) suggests that a large part of the subset of unique conserved plant genes may come from DNA transfer from the chloroplast to the nuclear genome. Horizontal transfer from bacteria to plant genome can also explain a fraction of this gene subset. This gene transfer probably predated the speciation between *Liliopsida *and core eudicotyledons for the concerned U{1:1} genes and is close to the root of the plant phylum for the group of U{1:1:1:1} genes. Our results suggest that, after their transfer to the nucleus, these genes have been submitted to a strong selection pressure that conserved them as unique. This hypothesis is more parsimonious than many independent gene transfer events in each concerned plant species. In their 26 clusters of pan-orthologs, Zimmer *et al*. [78] also suggest a DNA transfer from organellar genome, mainly from mitochondria. Our observations on the U{1:1} gene population showed that transfer from mitochondria was also significant (Chi-squared test, P-value = 0.0002) but less important than from chloroplasts (Table 5).

**Table 5:** Phylogenetic profile, subcellular localization and promoter of U{1:1} genes and proteins

		Predicted targeting				Promoter	
			
	Gene number	Plastid	Mito.	Nucleus	ER	TATA	TELO
**Random nuclear genes**	**20,000**	**6.0%**	**3.6%**	**5.6%**	**14.7%**	**19.9%**	**12.2%**
plant	50.5%	4.0%	3.1%	6.3%	15.5%	22.1%	9.2%
plant + bacteria	5.5%	28.5%	3.5%	1.8%	11.6%	14.6%	5.7%
plant + metazoa	20.4%	1.9%	2.4%	7.7%	8.0%	16.5%	20.5%
plant + bacteria + metazoa	23.6%	8.4%	5.7%	3.2%	19.4%	20.7%	10.7%

**U{1:1} genes**	**937**	**15.5%**	**6.0%**	**4.9%**	**6.8%**	**10.3%**	**15.2%**
plant	49.7%	16.5%	5.4%	5.8%	8.1%	8.4%	16.2%
plant + bacteria	11.3%	49.1%	3.8%	1.0%	2.8%	17.7%	4.4%
plant + metazoa	27.9%	1.2%	4.6%	6.1%	6.5%	8.2%	18.5%
plant + bacteria + metazoa	11.1%	12.5%	14.4%	1.9%	5.8%	18.0%	11.5%

Phylum conservation of 937 U{1:1} genes and 20,000 random nuclear genes was obtained by BLASTp of conserved unique *A. thaliana *proteins against the Uniprot database. Gene number (column 1) shows the relative number of genes with a sequence similarity suggesting homology in different phyla. Subcellular localization (column 2–4) was retrieved from the FLAGdb^++ ^database. ER means Endoplasmic Reticulum. Promoter regions of the *A. thaliana *genes were analysed for the presence of TATA (column 5) and TELO (column 6) boxes as previously described in Table 1 and the Methods section.

A second subset of U{1:1} genes with homologs in metazoa (including fungi) must have been conserved from ancient eukaryotic cells through the entire phylum and probably has a critical function. Ancient origin, low divergence rate, presence of TELO-box and dearth of TATA-box (Table 5), suggest that they are, or are related to, housekeeping genes [47,84] but no evidence could be retrieved from the Gene Ontology annotation due to the high number of unclassified genes. This metazoan conserved subset represents 28% of the 937 U{1:1} genes but, interestingly, this fraction increases to 43% in the 192 U{1:1:1:1} genes.

## Conclusion

We defined 2,570 and 8,041 proteins as unique in *A. thaliana *and *O. sativa *respectively. Unique proteins, products of unique (or single-copy) genes, are proteins with no sequence motif shared by any other protein in the same species. *A. thaliana *unique genes can be further classified according to the number of orthologous genes found in *O. sativa *genome or *vice-versa*. Final classification included: 451 AtU{1:0}NE, 544 AtU{1:0}E, 974 At U{1:1}, 601 AtU{1:m}, 4956 OsU{1:0}NE, 1462 OsU{1:0}E, 960 OsU{1:1} and 663 OsU{1:m} genes (2).

Unique genes are distributed all over the genomes including regions with evidence for segmental duplication and suggesting that unique genes have been created by non-reciprocal local losses between two paralogous duplicated genomic regions. These non-reciprocal losses may have been directed by a selective pressure according to the structural features present in unique genes conserved in the two species (U{1:1} and U{1:m} genes). These specific features are a relatively small protein size and a high intron density that have been described as evidence of a slow evolution rate [43]. From a functional point of view, unique conserved genes are characterized by a rare occurrence of TATA-box and a high occurrence of TELO-box in their promoters suggesting that unique genes could be linked to critical housekeeping functions such as protein catabolism and synthesis, RNA processing or DNA repair [47,71,84]. These results differ from previous observations which showed that genes involved in transcription regulation and signal transduction tend to be more duplicated [12,85]. Additionally, even if unique genes have been conserved in plants, no significant over-representation of TFBS related with photosynthesis or light regulation processes, such as SORLIP2 and CAAT boxes, have been found in *A. thaliana *and *O. sativa *(Table 1).

Unlike conserved single copy genes, the *A. thaliana *and *O. sativa *U{1:0} genes exhibit a low intron density, a normal presence of TFBS in their promoters, and they encode for proteins about 2.5 times shorter when compared to all the nuclear genes. Very short proteins have been reported as proproteins, precursors of regulatory peptides [86]. Despite the fact that the function of 80% of AtU{1:0}E genes and 95% of OsU{1:0}E genes remains unknown, the analysis of the 105 AtU{1:0}E with annotated function seems to reinforce this hypothesis as we have found that many AtU{1:0}E code for known precursors of short peptide phytohormones with signalling roles [61].

From a phylogenetic point of view, product length conservation and similar relative transcription level of the 937 pan-orthologous genes in *A. thaliana *and *O. sativa *(U{1:1}) are clear evidence of a common origin. However, intron insertion site conservation is the best proof that couples of U{1.1} have evolved from a common ancestor and are not the consequence of convergence. This intron conservation is also evident for the 192 U{1:1:1:1} genes where dN/dS analysis shows that those genes conserved as unique in very distant photosynthetic species are pan-orthologs under negative selection pressure to keep them in a low divergence rate and unique. This situation reinforces the idea of a probable important conserved function.

It could be suggested that the characterization of pan-orthologs (conserved single copy genes in two or more species) could be noised by the presence of paralogs in the situation where opposite members of a pair of duplicated genes are lost in two daughter species. Nevertheless, our results about conservation of protein sizes, transcription levels and sequence conservation (dN/dS) argue that, if it is the case, the gene loss occurred before both duplicates diverged enough to allow us to recognized them as paralogs rather than as orthologs.

The phylogenetic profiles of conserved single copy genes and the predicted subcellular location of the corresponding proteins, provides additional information on the origin and the function of these particular genes. An *A. thaliana *subset of unique genes with homologs in plants and bacteria contains 49.1% of genes encoding proteins with targeting peptides specific to the chloroplast. This observation suggests that the origin of this subset of unique genes could be a DNA transfer from chloroplast or bacteria genome posterior to the eukaryote radiation.

Our analysis of the conserved single copy genes, coming in addition to many duplicated gene studies, provides new information on plant gene evolution. Thus, an important part of the genes in only one copy in present plant genomes have an ancient origin and a low divergence rate controlled by a strong selection pressure. The species-specific unique genes that have some structural features in common with the conserved single copy genes are probably recruited from some conserved single copy genes experiencing a rapid divergence linked to a speciation event. However, functions of many of these conserved single copy genes remain unknown. Deeper annotation of small coding sequences that may not be identified by gene finders because of the conservative nature of the prediction algorithms, as well as more experimental data could help to decipher the biological functions of this particular gene population.

## Methods

### Data sources

The complete proteomes were obtained from TAIR [87] for *A. thaliana *(R6), TIGR [88] for *O. sativa *(R3), and JGI [89] for *P. patens *and *O. lucimarinus*. For *A. thaliana *and *O. sativa*, we retrieved data concerning the number of transcripts, the PFAM motifs and the promoter sequences from FLAGdb^++ ^[90]. Expression data were obtained from CATdb [37] and Genevestigator [39].

### Unique gene characterization

All the proteins encoded by the nuclear genes of each species were retrieved and those from pseudogenes were removed. To identify genes coding for proteins unique in a genome, three different filters were successively applied to the genes (Figure 1). The first filter used the PFAM resource [31] and was selected based on the fact that proteins with common protein motifs are most often homologs. The detection of PFAM motifs is based on HMM profiles (through the HMMER tool) which are more adapted than simple sequence comparisons for the definition of conserved regions, allowing us to eliminate paralogs. All the proteins without PFAM motifs were saved in a list of candidate unique proteins and those with PFAM motifs were re-filtered to select as candidates only the proteins for which the PFAM is unique in the analysed proteome. Second, the proteins encoded by candidate unique genes were compared against the whole proteome through BLASTp. Indeed, the fact that the PFAM resource does not tag around 30% of *A. thaliana *and *O. sativa *proteins and the risk that the PFAM filter introduces bias in tagging preferentially large proteins is corrected by additional BLAST analyses. Furthermore, we have taken care that our BLASTp parameters allow the detection of similarities between very small proteins: Proteins giving an e-value lower than e-10 with another protein in the same genome were discarded from the unique gene list. Third, the genes giving an e-value between e-5 and e-10 with another sequence were considered as unique genes only if they showed a partial match not larger than 30% of their sequence length (size ratio filter). This cut-off (size ratio filter), based on manual expertise of numerous blast results, permitted us to keep genes with hits too small to be considered as probably good despite the e-value obtained.

### Conserved single copy genes

A BLASTp of the unique proteins of each species was launched against a database containing the unique protein sequences from every other species. Pairs of proteins showing an e-value lower than e-10, or up to e-5 but satisfying the condition imposed by to the size ratio filter described above, were classified as conserved between the two species. Conserved proteins were then separated into two groups, the U{1:1} proteins if there was only one positive hit or the U{1:m} proteins if there were more than one hit. U{1:1} genes characterized in each species were compared to select only reciprocal best hits (RBH) and allowed us to remove some U{1:1} in one species qualified as U{1:m} in other species due to a splitting/fusion process. A second BLASTp was launched with those proteins without any hit against a database containing all the proteins from every other species. Applying again the same e-value and size ratio filter as described above, we clustered them as U{1:m} proteins if they had more than one hit, and as U{1:0} if they had no hit on the other species, *i.e*. the species specific unique proteins.

### Genomic organization of unique genes

The limits defining the boundaries of duplicated regions in *A. thaliana *and *O. sativa *genomes were retrieved from TIGR database [88]. The even distribution of each group of unique gene pairs between the chromosomes was tested using a chi-square (χ^2^) test with a confidence level of 99.5% (expected value of 14.86 and 26.76 for 4 and 11 degrees of freedom, respectively).

### Unique gene and protein features

All the different information about genes and proteins was retrieved from the FLAGdb^++ ^database [90]. Information includes protein lengths, number of exons, intron positions and promoter sequences (see Additional file 1). Only the genes with CDS fully covered by experimental transcript data were used (17,108 and 15,814 nuclear genes in *A. thaliana *and *O. sativa *respectively). For the analysis of promoter sequences, only genes with at least one cognate transcript covering the regions were studied (14,689 and 17,720 for *A. thaliana *and *O. sativa *respectively). Intron positions were compared after aligning protein sequences with ClustalW [91]. Intronic conserved positions included those that diverged by not more than 5 amino acids to take into account minor variability in intron position found in different organisms [92]. For promoter analyses, the TSS (Transcription Start Site) was defined as the point where the 5' UTR (minimum size of 50 bp) started and promoter sequences comprised the 1,000 nucleotides upstream from it. Positions of such well-known promoters as the TATA (TATAWA consensus [93]), TELO (AAACCCTAA consensus [47], SORLIP2 (also called motif II: GGCCA consensus [47,48]) and CAAT (CCAAT consensus [49]) boxes in each species were set with a program developed by Bernard *et al *[94] capable of defining significant TFBS preferential positions in promoter regions avoiding false positives [95]. If the TSS defines position 1, in *A. thaliana *preferential positions were set at: -40 to -21 for TATA-box; -60 to 140 for TELO-box; -240 to -21 for SORLIP2-box and -160 to -41 for CAAT-box. Similarly, in *O. sativa *TFBS were searched for in the following regions: -40 to -21 for TATA-box; -80 to 180 for TELO-box; -280 to -1 for SORLIP2-box and -200 to -1 for CAAT-box.

### At and Os U{1:1} gene expression

We based our estimation of the correlation between U{1:1}gene expression in *A. thaliana *and *O. sativa *on EST/cDNA resources. The numbers of associated transcripts of each gene were normalized and logarithmically transformed for comparisons purposes. Normalization avoided biases caused by both the number of transcripts available and the different number of genes for each species. The normalization established an equivalence of 1.56 transcripts in *O. sativa *for one transcript in *A. thaliana*. Comparisons of observed values were made against values from 100 random samples of 937 nuclear gene pairs. To avoid sampling biases due to genes with none or very few transcripts, we only considered the gene pairs with at least 30 cognate transcripts for each member. Furthermore, the random samples only contained protein pairs with a maximum size difference of 20 amino acids between the two members.

### Phylogenetic and functional analyses

The phylogenetic evolution of unique genes was analysed from *Ostreococcus lucimarinus *(Prasinophyceae) to *Arabidopsis thaliana *including *Physcomitrella patens *(Funariaceae) and *Oryza sativa*. With the unique gene characterization method (described above), we systematically searched for unique proteins in the available proteomes of the four species studied. Once obtained, we used them in a BLASTp search to look for *O. lucimarinus *unique proteins with a pan-ortholog on each branch of evolution (Figure 6). By this way, we first constructed U{1:1} protein pairs between *O. lucimarinus *and *P. patens*. After, *O. lucimarinus *U{1:1} proteins were used in a new BLASTp comparison against *O. sativa *unique proteins to found U{1:1:1} proteins, and so on until the characterization of the U{1:1:1:1} proteins. Similar protocol was performed starting from *A. thaliana *unique proteins and looking for their conservation on each node of the tree. Both lists of U{1:1:1:1} genes, one per sense, were crossed to eliminate inconsistencies and obtain a final list of 192 U{1:1:1:1} proteins. We calculated the expected conserved number of U{1:1:1:1} genes from *O. lucimarinus *to *A. thaliana *under the no selection pressure hypothesis. The expected number of U{1:1:1:1}genes assuming random conservation was calculated as the number of *O. lucimarinus *genes (7,618) multiplied by the combined probability of a gene being conserved as unique in *O. lucimarinus *(35.32%), *P. patens *(23.22%), *O. sativa *(13.88%) and *A. thaliana *(9.58%). The expected number of U{1:1:1:1} genes by random conservation would be 8.38 genes. For each species pair permutation, unique genes were aligned with their corresponding ortholog using ClustalW, and the synonymous and non-synonymous substitution rates (dN and dS) were calculated using the Codeml program of the PAML package [81]. The protein pairs considered as too divergent by Codeml were nevertheless taken into account in the median dN/dS calculation. For comparison, dN and dS values were also calculated with the same method from a set of 7,551 orthologous proteins predicted by the RBH method using the 3 proteomes of *A. thaliana, O. sativa *and *V. vinifera*. Conservation of U{1:1:1:1} genes in other species and functional information were retrieved from the results of BLASTp against the Uniprot database, with a limit e-value of e-10. Comparisons were done against the results of 100 random samples of 200 nuclear genes. The subcellular localization of *A. thaliana *proteins deduced from predictions of signal sequences (based on PSORT, PREDOTAR and CHLOROP software) were recovered from the FLAGdb^++ ^database [90]. The presence of cis-regulatory motifs within promoters was searched with the protocol previously described [94].

## Abbreviations

TFBS: Transcription Factor Binding Sites; MYA: Million Years Ago; RBH: Reciprocal Best Hit; TSS: Transcription Start Site; aa: amino acids.

## Authors' contributions

DA conducted the analyses and drafted the manuscript. AL and SA supervised the project, contributed to data interpretation and improved the manuscript. All authors have read and approved the final version of the manuscript.

## Supplementary Material

Additional file 1 **Additional Table1**. Information about the 937 U{1:1} genes and proteins. Each line corresponds to a couple of orthologous genes. 1. Id. of the *A. thaliana *gene. 2. Id. of the *O. sativa *gene. 3. Size of the protein encoded by the *A. thaliana *gene (in aa). Shaded in: Grey, genes with CDS fully covered by ESTs/cDNA. 4. Size of the protein encoded by the *O. sativa *gene (in aa). Shaded in: Grey, genes with CDS fully covered by ESTs/cDNA. 5. Number of exons of *A. thaliana *gene. Shaded in: Grey, genes with CDS fully covered by ESTs/cDNA. 6. Number of exons of *O. sativa *gene. Shaded in: Grey, genes with CDS fully covered by ESTs/cDNA. 7. Percentage of similarity between *A. thaliana *and *O. sativa *proteins after a ClustalW alignment. 8. Protein targeting according to predicted signal peptide in *A. thaliana*. Shaded in: Light Red: mitochondria; Blue: nucleus; Light Purple: endoplasmatic reticulum; Light Green: plastid. 9. Protein targeting according to predicted signal peptide in *O. sativa*. Shaded in: Light Red: mitochondria; Blue: nucleus; Light Purple: endoplasmatic reticulum; Light Green: plastid. 10. TATA-box within the promoter sequence of the *A. thaliana *gene. Shaded in: Red: no presence; Green: presence. 11. TATA-box within the promoter sequence of the *O. sativa *gene. Shaded in: Red: no presence; Green: presence. 12. TELO-box within the promoter sequence of the *A. thaliana *gene. Shaded in: Red: no presence; Green: presence. 13. TELO-box within the promoter sequence of the *O. sativa *gene. Shaded in: Red: no presence; Green: presence. 14. SORLIP2-box within the promoter sequence of the *A. thaliana *gene. Shaded in: Red: no presence; Green: presence. 15. SORLIP2-box within the promoter sequence of the *O. sativa *gene. Shaded in: Red: no presence; Green: presence. 16. CAAT-box within the promoter sequence of the *A. thaliana *gene. Shaded in: Red: no presence; Green: presence. 17. CAAT-box within the promoter sequence of the *O. sativa *gene. Shaded in: Red: no presence; Green: presence. 18. Gene conservation in *P. patens *according to BLASTp results. Shaded in: Green: conservation of both species genes; Yellow: conservation of *A. thaliana *gene; Blue: conservation of *O. sativa *gene. 19. Gene conservation in *O. lucimarinus *according to BLASTp results. Shaded in: Green: conservation of both species genes; Yellow: conservation of *A. thaliana *gene; Blue: conservation of *O. sativa *gene. 20. Phylogenetic conservation of genes in plant, bacteria and metazoa taxa. Results based on BLASTp of *A. thaliana *protein sequence against the Uniprot database. 21. Gene function based on annotated functions retrieved from BLASTp results on the Uniprot database. Click here for file

## References

[B1] Taylor JS Raes J Duplication and divergence: the evolution of new genes and old ideas Annu Rev Genet 2004 38 615 643 10.1146/annurev.genet.38.072902.092831 15568988

[B2] Tekaia F Dujon B Pervasiveness of gene conservation and persistence of duplicates in cellular genomes J Mol Evol 1999 49 5 591 600 10.1007/PL00006580 10552040

[B3] Wapinski I Pfeffer A Friedman N Regev A Natural history and evolutionary principles of gene duplication in fungi Nature 2007 449 7158 54 61 10.1038/nature06107 17805289

[B4] The Arabidopsis Genome Initiative Analysis of the genome sequence of the flowering plant Arabidopsis thaliana Nature 2000 408 6814 796 815 10.1038/35048692 11130711

[B5] Yu J Hu S Wang J Wong GK Li S Liu B Deng Y Dai L Zhou Y Zhang X A draft sequence of the rice genome (Oryza sativa L. ssp. indica) Science 2002 296 5565 79 92 10.1126/science.1068037 11935017

[B6] Lynch M Conery JS The evolutionary fate and consequences of duplicate genes Science 2000 290 5494 1151 1155 10.1126/science.290.5494.1151 11073452

[B7] Koszul R Caburet S Dujon B Fischer G Eucaryotic genome evolution through the spontaneous duplication of large chromosomal segments Embo J 2004 23 1 234 243 10.1038/sj.emboj.7600024 14685272 PMC1271662

[B8] Holland PW Vertebrate evolution: something fishy about Hox genes Curr Biol 1997 7 9 R570 572 10.1016/S0960-9822(06)00284-3 9285709

[B9] Pebusque MJ Coulier F Birnbaum D Pontarotti P Ancient large-scale genome duplications: phylogenetic and linkage analyses shed light on chordate genome evolution Mol Biol Evol 1998 15 9 1145 1159 9729879 10.1093/oxfordjournals.molbev.a026022

[B10] Spring J Vertebrate evolution by interspecific hybridisation – are we polyploid? FEBS Lett 1997 400 1 2 8 10.1016/S0014-5793(96)01351-8 9000502

[B11] Simillion C Vandepoele K Van Montagu MC Zabeau M Peer Y Van de The hidden duplication past of Arabidopsis thaliana Proc Natl Acad Sci USA 2002 99 21 13627 13632 10.1073/pnas.212522399 12374856 PMC129725

[B12] Blanc G Wolfe KH Widespread paleopolyploidy in model plant species inferred from age distributions of duplicate genes Plant Cell 2004 16 7 1667 1678 10.1105/tpc.021345 15208399 PMC514152

[B13] Paterson AH Bowers JE Chapman BA Ancient polyploidization predating divergence of the cereals, and its consequences for comparative genomics Proc Natl Acad Sci USA 2004 101 26 9903 9908 10.1073/pnas.0307901101 15161969 PMC470771

[B14] Rubin GM Yandell MD Wortman JR Gabor Miklos GL Nelson CR Hariharan IK Fortini ME Li PW Apweiler R Fleischmann W Comparative genomics of the eukaryotes Science 2000 287 5461 2204 2215 10.1126/science.287.5461.2204 10731134 PMC2754258

[B15] Llorente B Durrens P Malpertuy A Aigle M Artiguenave F Blandin G Bolotin-Fukuhara M Bon E Brottier P Casaregola S Genomic exploration of the hemiascomycetous yeasts: 20. Evolution of gene redundancy compared to Saccharomyces cerevisiae FEBS Lett 2000 487 1 122 133 10.1016/S0014-5793(00)02291-2 11152895

[B16] Lynch M Conery JS The evolutionary demography of duplicate genes J Struct Funct Genomics 2003 3 1–4 35 44 10.1023/A:1022696612931 12836683

[B17] Scannell DR Frank AC Conant GC Byrne KP Woolfit M Wolfe KH Independent sorting-out of thousands of duplicated gene pairs in two yeast species descended from a whole-genome duplication Proc Natl Acad Sci USA 2007 104 20 8397 8402 10.1073/pnas.0608218104 17494770 PMC1895961

[B18] Kondrashov FA Rogozin IB Wolf YI Koonin EV Selection in the evolution of gene duplications Genome Biol 2002 3 2 RESEARCH0008 10.1186/gb-2002-3-2-research0008 11864370 PMC65685

[B19] Maere S De Bodt S Raes J Casneuf T Van Montagu M Kuiper M Peer Y Van de Modeling gene and genome duplications in eukaryotes Proc Natl Acad Sci USA 2005 102 15 5454 5459 10.1073/pnas.0501102102 15800040 PMC556253

[B20] Seoighe C Wolfe KH Updated map of duplicated regions in the yeast genome Gene 1999 238 1 253 261 10.1016/S0378-1119(99)00319-4 10571001

[B21] Krylov DM Wolf YI Rogozin IB Koonin EV Gene loss, protein sequence divergence, gene dispensability, expression level, and interactivity are correlated in eukaryotic evolution Genome Res 2003 13 10 2229 2235 10.1101/gr.1589103 14525925 PMC403683

[B22] Gaillardin C Duchateau-Nguyen G Tekaia F Llorente B Casaregola S Toffano-Nioche C Aigle M Artiguenave F Blandin G Bolotin-Fukuhara M Genomic exploration of the hemiascomycetous yeasts: 21. Comparative functional classification of genes FEBS Lett 2000 487 1 134 149 10.1016/S0014-5793(00)02292-4 11152896

[B23] Clauss MJ Mitchell-Olds T Functional divergence in tandemly duplicated Arabidopsis thaliana trypsin inhibitor genes Genetics 2004 166 3 1419 1436 10.1534/genetics.166.3.1419 15082560 PMC1470761

[B24] Lawton-Rauh A Evolutionary dynamics of duplicated genes in plants Mol Phylogenet Evol 2003 29 3 396 409 10.1016/j.ympev.2003.07.004 14615182

[B25] Gutierrez RA Green PJ Keegstra K Ohlrogge JB Phylogenetic profiling of the Arabidopsis thaliana proteome: what proteins distinguish plants from other organisms? Genome Biol 2004 5 8 R53 10.1186/gb-2004-5-8-r53 15287975 PMC507878

[B26] Gollery M Harper J Cushman J Mittler T Girke T Zhu JK Bailey-Serres J Mittler R What makes species unique? The contribution of proteins with obscure features Genome Biol 2006 7 7 R57 10.1186/gb-2006-7-7-r57 16859532 PMC1779552

[B27] Chothia C Gough J Vogel C Teichmann SA Evolution of the protein repertoire Science 2003 300 5626 1701 1703 10.1126/science.1085371 12805536

[B28] Wolfe KH Gouy M Yang YW Sharp PM Li WH Date of the monocot-dicot divergence estimated from chloroplast DNA sequence data Proc Natl Acad Sci USA 1989 86 16 6201 6205 10.1073/pnas.86.16.6201 2762323 PMC297805

[B29] Chaw SM Chang CC Chen HL Li WH Dating the monocot-dicot divergence and the origin of core eudicots using whole chloroplast genomes J Mol Evol 2004 58 4 424 441 10.1007/s00239-003-2564-9 15114421

[B30] Blair JE Shah P Hedges SB Evolutionary sequence analysis of complete eukaryote genomes BMC Bioinformatics 2005 6 53 10.1186/1471-2105-6-53 15762985 PMC1274250

[B31] Bateman A Coin L Durbin R Finn RD Hollich V Griffiths-Jones S Khanna A Marshall M Moxon S Sonnhammer EL The Pfam protein families database Nucleic Acids Research 2004 32 D138 D141 10.1093/nar/gkh121 14681378 PMC308855

[B32] Altschul SF Gish W Miller W Myers EW Lipman DJ Basic local alignment search tool J Mol Biol 1990 215 3 403 410 2231712 10.1016/S0022-2836(05)80360-2

[B33] Sterck L Rombauts S Vandepoele K Rouzé P Peer Y Van de How many genes are there in plants (and why are they there)? Current Opinion in Plant Biology 2007 10 2 199 203 10.1016/j.pbi.2007.01.004 17289424

[B34] Mohseni-Zadeh S Louis A Brezellec P Risler JL PHYTOPROT: a database of clusters of plant proteins Nucleic Acids Research 2004 32 Database D351 D353 10.1093/nar/gkh040 14681432 PMC308774

[B35] Wu F Mueller LA Crouzillat D Petiard V Tanksley SD Combining bioinformatics and phylogenetics to identify large sets of single-copy orthologous genes (COSII) for comparative, evolutionary and systematic studies: a test case in the euasterid plant clade Genetics 2006 174 3 1407 1420 10.1534/genetics.106.062455 16951058 PMC1667096

[B36] Glemet E Codani JJ LASSAP, a LArge Scale Sequence compArison Package Comput Appl Biosci 1997 13 2 137 143 9146960 10.1093/bioinformatics/13.2.137

[B37] Gagnot S Tamby JP Martin-Magniette ML Bitton F Taconnat L Balzergue S Aubourg S Renou JP Lecharny A Brunaud V CATdb: a public access to Arabidopsis transcriptome data from the URGV-CATMA platform Nucleic Acids Res 2008 36 Database D986 990 17940091 10.1093/nar/gkm757 PMC2238931

[B38] Zimmermann P Hennig L Gruissem W Gene-expression analysis and network discovery using Genevestigator Trends Plant Sci 2005 10 9 407 409 10.1016/j.tplants.2005.07.003 16081312

[B39] Zimmermann P Hirsch-Hoffmann M Hennig L Gruissem W GENEVESTIGATOR. Arabidopsis microarray database and analysis toolbox Plant Physiol 2004 136 1 2621 2632 10.1104/pp.104.046367 15375207 PMC523327

[B40] Guyot R Keller B Ancestral genome duplication in rice Genome 2004 47 610 614 10.1139/g04-016 15190378

[B41] The Rice Chromosomes 11 and 12 Sequencing Consortia The sequence of rice chromosomes 11 and 12, rich in disease resistance genes and recent gene duplications BMC Biology 2005 3 20 10.1186/1741-7007-3-20 16188032 PMC1261165

[B42] Yu J Wang J Lin W Li S Li H Zhou J Ni P Dong W Hu S Zeng C The Genomes of Oryza sativa: a history of duplications PLoS Biol 2005 3 2 e38 10.1371/journal.pbio.0030038 15685292 PMC546038

[B43] Carmel L Rogozin IB Wolf YI Koonin EV Evolutionarily conserved genes preferentially accumulate introns Genome Res 2007 17 7 1045 1050 10.1101/gr.5978207 17495009 PMC1899115

[B44] Brooks AR Nagy BP Taylor S Simonet WS Taylor JM Levy-Wilson B Sequences containing the second-intron enhancer are essential for transcription of the human apolipoprotein B gene in the livers of transgenic mice Mol Cell Biol 1994 14 4 2243 2256 8139530 10.1128/mcb.14.4.2243-2256.1994 PMC358591

[B45] Carmel L Wolf YI Rogozin IB Koonin EV Three distinct modes of intron dynamics in the evolution of eukaryotes Genome Res 2007 17 7 1034 1044 10.1101/gr.6438607 17495008 PMC1899114

[B46] Benabdellah K Gonzalez-Rey E Gonzalez A Alternative trans-splicing of the Trypanosoma cruzi LYT1 gene transcript results in compartmental and functional switch for the encoded protein Mol Microbiol 2007 65 6 1559 1567 10.1111/j.1365-2958.2007.05892.x 17824931

[B47] Tremousaygue D Garnier L Bardet C Dabos P Herve C Lescure B Internal telomeric repeats and 'TCP domain' protein-binding sites co-operate to regulate gene expression in Arabidopsis thaliana cycling cells Plant J 2003 33 6 957 966 10.1046/j.1365-313X.2003.01682.x 12631321

[B48] Hudson ME Quail PH Identification of promoter motifs involved in the network of phytochrome A-regulated gene expression by combined analysis of genomic sequence and microarray data Plant Physiol 2003 133 4 1605 1616 10.1104/pp.103.030437 14681527 PMC300717

[B49] Bucher P Trifonov EN CCAAT box revisited: bidirectionality, location and context J Biomol Struct Dyn 1988 5 6 1231 1236 3271510 10.1080/07391102.1988.10506466

[B50] Skovgaard M Jensen LJ Brunak S Ussery D Krogh A On the total number of genes and their length distribution in complete microbial genomes Trends Genet 2001 17 8 425 428 10.1016/S0168-9525(01)02372-1 11485798

[B51] Linial M How incorrect annotations evolve – the case of short ORFs Trends Biotechnol 2003 21 7 298 300 10.1016/S0167-7799(03)00139-2 12837613

[B52] Snyder M Gerstein M Genomics. Defining genes in the genomics era Science 2003 300 5617 258 260 10.1126/science.1084354 12690176

[B53] Lease KA Walker JC The Arabidopsis unannotated secreted peptide database, a resource for plant peptidomics Plant Physiol 2006 142 3 831 838 10.1104/pp.106.086041 16998087 PMC1630735

[B54] Hanada K Zhang X Borevitz JO Li WH Shiu SH A large number of novel coding small open reading frames in the intergenic regions of the Arabidopsis thaliana genome are transcribed and/or under purifying selection Genome Res 2007 17 5 632 640 10.1101/gr.5836207 17395691 PMC1855179

[B55] Aubourg S Martin-Magniette ML Brunaud V Taconnat L Bitton F Balzergue S Jullien PE Ingouff M Thareau V Schiex T Analysis of CATMA transcriptome data identifies hundreds of novel functional genes and improves gene models in the Arabidopsis genome BMC Genomics 2007 8 401 10.1186/1471-2164-8-401 17980019 PMC2174955

[B56] Moskal WA Jr Wu HC Underwood BA Wang W Town CD Xiao Y Experimental validation of novel genes predicted in the un-annotated regions of the Arabidopsis genome BMC Genomics 2007 8 18 10.1186/1471-2164-8-18 17229318 PMC1783852

[B57] Frith MC Forrest AR Nourbakhsh E Pang KC Kai C Kawai J Carninci P Hayashizaki Y Bailey TL Grimmond SM The abundance of short proteins in the mammalian proteome PLoS Genet 2006 2 4 e52 10.1371/journal.pgen.0020052 16683031 PMC1449894

[B58] Kastenmayer JP Ni L Chu A Kitchen LE Au WC Yang H Carter CD Wheeler D Davis RW Boeke JD Functional genomics of genes with small open reading frames (sORFs) in S. cerevisiae Genome Res 2006 16 3 365 373 10.1101/gr.4355406 16510898 PMC1415214

[B59] Galindo MI Pueyo JI Fouix S Bishop SA Couso JP Peptides encoded by short ORFs control development and define a new eukaryotic gene family PLoS Biol 2007 5 5 e106 10.1371/journal.pbio.0050106 17439302 PMC1852585

[B60] Swarbreck D Wilks C Lamesch P Berardini TZ Garcia-Hernandez M Foerster H Li D Meyer T Muller R Ploetz L The Arabidopsis Information Resource (TAIR): gene structure and function annotation Nucleic Acids Res 2008 36 Database D1009 1014 17986450 10.1093/nar/gkm965 PMC2238962

[B61] Farrokhi N Whitelegge JP Brusslan JA Plant peptides and peptidomics Plant Biotechnol J 2008 6 2 105 134 10.1111/j.1467-7652.2007.00315.x 18069950

[B62] Tatusov RL Koonin EV Lipman DJ A genomic perspective on protein families Science 1997 278 5338 631 637 10.1126/science.278.5338.631 9381173

[B63] Knowles DG McLysaght A High rate of recent intron gain and loss in simultaneously duplicated Arabidopsis genes Mol Biol Evol 2006 23 8 1548 1557 10.1093/molbev/msl017 16720694

[B64] Roy SW Penny D Patterns of intron loss and gain in plants: Intron loss-dominated evolution and genome-wide comparison of O. sativa and A. thaliana Molecular Biology and Evolution 2007 24 1 171 180 10.1093/molbev/msl159 17065597

[B65] Pal C Papp B Hurst LD Highly expressed genes in yeast evolve slowly Genetics 2001 158 2 927 931 11430355 10.1093/genetics/158.2.927 PMC1461684

[B66] Drummond DA Raval A Wilke CO A single determinant dominates the rate of yeast protein evolution Mol Biol Evol 2006 23 2 327 337 10.1093/molbev/msj038 16237209

[B67] Wright SI Yau CB Looseley M Meyers BC Effects of gene expression on molecular evolution in Arabidopsis thaliana and Arabidopsis lyrata Mol Biol Evol 2004 21 9 1719 1726 10.1093/molbev/msh191 15201397

[B68] Berezikov E Guryev V Cuppen E CONREAL web server: identification and visualization of conserved transcription factor binding sites Nucleic Acids Research 2005 33 1 W447 W450 10.1093/nar/gki378 15980509 PMC1160139

[B69] Hindemitt T Mayer KF CREDO: a web-based tool for computational detection of conserved sequence motifs in noncoding sequences Bioinformatics 2005 21 23 4304 4306 10.1093/bioinformatics/bti691 16204349

[B70] Thijs G Lescot M Marchal K Rombauts S De Moor B Rouze P Moreau Y A higher-order background model improves the detection of promoter regulatory elements by Gibbs sampling Bioinformatics 2001 17 12 1113 1122 10.1093/bioinformatics/17.12.1113 11751219

[B71] Walther D Brunnemann R Selbig J The regulatory code for transcriptional response diversity and its relation to genome structural properties in A. thaliana PLoS Genet 2007 3 2 e11 10.1371/journal.pgen.0030011 17291162 PMC1796623

[B72] Moshonov S Elfakess R Golan-Mashiach M Sinvani H Dikstein R Links between core promoter and basic gene features influence gene expression BMC Genomics 2008 9 1 92 10.1186/1471-2164-9-92 18298820 PMC2279122

[B73] Palenik B Grimwood J Aerts A Rouze P Salamov A Putnam N Dupont C Jorgensen R Derelle E Rombauts S The tiny eukaryote Ostreococcus provides genomic insights into the paradox of plankton speciation Proc Natl Acad Sci USA 2007 104 18 7705 7710 10.1073/pnas.0611046104 17460045 PMC1863510

[B74] Rensing SA Lang D Zimmer AD Terry A Salamov A Shapiro H Nishiyama T Perroud PF Lindquist EA Kamisugi Y The Physcomitrella genome reveals evolutionary insights into the conquest of land by plants Science 2008 319 5859 64 69 10.1126/science.1150646 18079367

[B75] Tuskan GA Difazio S Jansson S Bohlmann J Grigoriev I Hellsten U Putnam N Ralph S Rombauts S Salamov A The genome of black cottonwood, Populus trichocarpa (Torr. & Gray) Science 2006 313 5793 1596 1604 10.1126/science.1128691 16973872

[B76] Jaillon O Aury JM Noel B Policriti A Clepet C Casagrande A Choisne N Aubourg S Vitulo N Jubin C The grapevine genome sequence suggests ancestral hexaploidization in major angiosperm phyla Nature 2007 449 7161 463 467 10.1038/nature06148 17721507

[B77] Vandepoele K Peer Y Van de Exploring the plant transcriptome through phylogenetic profiling Plant Physiol 2005 137 1 31 42 10.1104/pp.104.054700 15644465 PMC548836

[B78] Zimmer A Lang D Richardt S Frank W Reski R Rensing SA Dating the early evolution of plants: detection and molecular clock analyses of orthologs Mol Genet Genomics 2007 278 4 393 402 10.1007/s00438-007-0257-6 17593393

[B79] Hedges SB Blair JE Venturi ML Shoe JL A molecular timescale of eukaryote evolution and the rise of complex multicellular life BMC Evol Biol 2004 4 2 10.1186/1471-2148-4-2 15005799 PMC341452

[B80] Nei M Gojobori T Simple methods for estimating the numbers of synonymous and nonsynonymous nucleotide substitutions Mol Biol Evol 1986 3 5 418 426 3444411 10.1093/oxfordjournals.molbev.a040410

[B81] Yang Z PAML 4: Phylogenetic Analysis by Maximum Likelihood Mol Biol Evol 2007 24 8 1586 1591 10.1093/molbev/msm088 17483113

[B82] Nekrutenko A Makova KD Li WH The K(A)/K(S) ratio test for assessing the protein-coding potential of genomic regions: an empirical and simulation study Genome Res 2002 12 1 198 202 10.1101/gr.200901 11779845 PMC155263

[B83] Anisimova M Bielawski JP Yang Z Accuracy and power of the likelihood ratio test in detecting adaptive molecular evolution Mol Biol Evol 2001 18 8 1585 1592 11470850 10.1093/oxfordjournals.molbev.a003945

[B84] Basehoar AD Zanton SJ Pugh BF Identification and distinct regulation of yeast TATA box-containing genes Cell 2004 116 5 699 709 10.1016/S0092-8674(04)00205-3 15006352

[B85] Seoighe C Gehring C Genome duplication led to highly selective expansion of the Arabidopsis thaliana proteome Trends Genet 2004 20 10 461 464 10.1016/j.tig.2004.07.008 15363896

[B86] Lindsey K Casson S Chilley P Peptides: new signalling molecules in plants Trends Plant Sci 2002 7 2 78 83 10.1016/S1360-1385(01)02194-X 11832279

[B87] TAIR http://www.arabidopsis.org/

[B88] TIGR http://www.tigr.org

[B89] JGI http://genome.jgi-psf.org/

[B90] Samson F Brunaud V Duchene S De Oliveira Y Caboche M Lecharny A Aubourg S FLAGdb++: a database for the functional analysis of the Arabidopsis genome Nucleic Acids Research 2004 32 Database D347 D350 10.1093/nar/gkh134 14681431 PMC308868

[B91] Chenna R Sugawara H Koike T Lopez R Gibson TJ Higgins DG Thompson JD Multiple sequence alignment with the Clustal series of programs Nucleic Acids Research 2003 31 13 3497 3500 10.1093/nar/gkg500 12824352 PMC168907

[B92] Boudet N Aubourg S Toffano-Nioche C Kreis M Lecharny A Evolution of intron/exon structure of DEAD helicase family genes in Arabidopsis, Caenorhabditis, and Drosophila Genome Res 2001 11 12 2101 2114 10.1101/gr.200801 11731501 PMC311229

[B93] Lifton RP Goldberg ML Karp RW Hogness DS The organization of the histone genes in Drosophila melanogaster: functional and evolutionary implications Cold Spring Harb Symp Quant Biol 1978 42 Pt 2 1047 1051 98262 10.1101/sqb.1978.042.01.105

[B94] Bernard V Brunaud V Serizet C Martin-Magniette ML Caboche M Aubourg S Lecharny A Sélection de motifs candidats pour la régulation des gènes chez *Arabidopsis thaliana *sur des critères topologiques JOBIM: 5–7 July 2006; Bordeaux 2006 17 28 ftp://urgv.evry.inra.fr/Publications/BernardV_et_al_JOBIM_5to7juli2006_Bordeaux_2006_17-28.pdf

[B95] Yamamoto YY Ichida H Matsui M Obokata J Sakurai T Satou M Seki M Shinozaki K Abe T Identification of plant promoter constituents by analysis of local distribution of short sequences BMC Genomics 2007 8 67 10.1186/1471-2164-8-67 17346352 PMC1832190

